# Highly Efficient Photocatalysts for Methylene Blue Degradation Based on a Platform of Deposited GO-ZnO Nanoparticles on Polyurethane Foam

**DOI:** 10.3390/molecules28010108

**Published:** 2022-12-23

**Authors:** Mohamed Morsy, Ahmed I. Abdel-Salam, Islam Gomaa, Hesham Moustafa, Haitham Kalil, Ahmed Helal

**Affiliations:** 1 Building Physics and Environment Institute, Housing & Building National Research Center (HBRC), Dokki, Giza 12311, Egypt; 2 Nanotechnology Research Centre (NTRC), The British University in Egypt (BUE), Suez Desert Road, El-Sherouk City, Cairo 11837, Egypt; 3 Department of Polymer Metrology & Technology, National Institute of Standards (NIS), Tersa Street, El Ha-ram, P.O. Box 136, Giza 12211, Egypt; 4 Bioanalysis Laboratory, National Institute of Standards (NIS), Tersa Street, El Haram, P.O. Box 136, Giza 12211, Egypt; 5 Chemistry Department, Cleveland State University, Cleveland, OH 44115, USA; 6 Chemistry Department, Faculty of Science, Suez Canal University, Ismailia 41522, Egypt; 7 Nanostructured Materials and Nanotechnology Division, Central Metallurgical Research and Development Institute, Helwan, Cairo 11722, Egypt

**Keywords:** photodegradation, methylene blue, ZnO nanoparticles, PUF/rGO/ZnO composite

## Abstract

The demand for reactive dyes in industries has increased rapidly in recent years, and producing a large quantity of dye-containing effluent waste contaminates soils and water streams. Current efforts to remove these harmful dyes have focused on utilizing functionalized nanomaterials. A 3D polyurethane foam loaded with reduced graphene oxide (rGO) and ZnO nanocomposite (PUF/rGO/ZnO) has been proposed as an efficient structural design for dye degradation under the influence of visible light. The proposed structure was synthesized using a hydrothermal route followed by microwave irradiation. The resultant 3D PUF/rGO/ZnO was examined and characterized by various techniques such as XRD, FTIR, SEM, EDAX, BET, and UV–visible spectroscopy. SEM data illustrated that a good dispersion and embedment of the rGO/ZnO NPs within the PUF matrix occurred. The adsorption capacity for neat PUF showed that around 20% of the Methylene blue (MB) dye was only adsorbed on its surface. However, it was found that an exceptional adsorption capacity for MB degradation was observed when the rGO/ZnO NPs inserted into the PUF, which initially deteriorated to ~ 70 % of its initial concentration. Notably, the MB dye was completely degraded within 3 h.

## 1. Introduction

With the vast growth of modern industry has come an increased awareness of the problem of water pollution [1,2]. Thus, uncounted efforts have been made to remove water pollution in terms of heavy metals and organic dyes using nanomaterials [3,4,5]. Recently, the demand for the utilization of reactive dyes in industries increased exponentially, thereby producing a large number of dye effluents as a byproduct [6]. These dye effluents are often discharged into the freshwater and soil without any pretreatment. All human beings are affected by effluents of dyes that contaminate water due to the lack of water reoxygenation, which inhibits sunlight and interferes with photosynthesis [7,8]. Dyes are water-soluble organic compounds with complex structures and high molecular weights. Traditional technologies for water treatments include physical, biological, and chemical methods [9]. Most of the utilized methods, when used to remove dyes, have the drawbacks of high cost, complicated technology, and the difficulty of completely removing dyes in a short period of time [10]. Efforts [11,12,13,14] have recently been conducted to develop more efficient, cost-effective photocatalysts for the removal of MB from contaminated water. 

Among these recent developments, polyurethane foams (PUFs), which have porous, flexible, and deformable skeletons, can be utilized when they are functionalized with materials for selective dye removal applications [15,16,17,18]. PUFs are widely used in various applications, such as fire retardant, antibacterial, and electromagnetically shielding substrates. In spite of these other applications, the use of PUF in water treatment is very limited [18]. This is due to the limited adhesion of inorganic pollutants to the surface of PUF [19]. Various functional nanoparticles, such as carbon nanotubes (CNTs) [20,21], graphene [22], and carbon black, have been proposed to be deposited over 3D PUFs in order to enhance their functionality [23]. Amongst, carbon-based nanomaterials, graphene is a 2D material with a good reputation in photocatalysts applications due to its outstanding electrical properties. However, the possibility of the agglomeration of graphene sheets via π–π interactions reduces the practical applications. As it is a superior conductive material, 2D graphene sheets have been deposited over PUF scaffolds to overcome the poor electrical conductivity and dye removal functionality of PU sponges [24]. Zinc oxide (ZnO) semiconductor material with wide band gaps (3.37 eV), high mechanical strength, and piezoelectric properties have also been explored for dye removal applications [25]. 1D ZnO nanorods have been explored as dye removal agents, and they have the advantages of low cost and high responsivity [26,27]. Liu et al. have studied the degradative activity of the Ag-ZnO-rGO ternary composite as a “green” photocatalyst. [28]. Their results have demonstrated that Ag-ZnO-rGO has a superior photocatalytic activity for the decomposition of methylene orange. This improved behavior was attributed to the suppression of the recombination of photogenerated charge carriers [29,30]. In synthesized core-shell rGO/ZnO, the effect of the rGO content on the efficiency of photodegradation of methylene blue was elucidated by Wang et al., who found that higher degradation efficiency was achieved under visible light due to the combination of rGO and ZnO. [31]. A novel ternary rGO-ZnO@Bi_2_MoO_6_ heterojunction has been synthesized by facile solvothermal methods and utilized for the photodegradation of rhodamine B (RhB) under irradiation by visible light. The results obtained demonstrate that this heterojunction’s photocatalytic efficiency was affected by the rGO ratio incorporated. The combination between ZnO and rGO led to a significant improvement in stability and photocatalytic activity, mostly due to effects on the recombination of photoexcited electrons, positive holes, decreasing band gap energy, reduced activation energy, and improved adsorption capacity [32,33]. Despite previous efforts, the proposed combination of PUF/rGO/ZnO has not, to the best of our knowledge, been reported. To take advantage of the photocatalytic merits of ZnO, the high electrical conductivity and abundance of hydroxyl functional group of rGO, as well as the 3D structure of PUF, the three materials have combined with the goal of improving dye removal from contaminated water [34]. Furthermore, the absorption of contaminated water into the 3D structure of PUF is promoted by this design, which further accelerates dye removal. In this study, the 3D PUF scaffold was loaded with rGO before the addition of ZnO. The 2D graphene sheets, as a superior conductive material, were deposited over the PU foam scaffold by impregnation under reduced pressure. The ZnO nanorods were then deposited over the PUF/rGO skeleton via impregnation and hydrothermal routes. The ZnO seed layer was deposited via an impregnation route, and the nanorods were then grown by a hydrothermal route. The characteristic features of the resulting structure of PUF/rGO/ZnO have been verified by XRD, FTIR, BET surface area, pore volume, and SEM-EDX measurements. The adsorption capacity of MB degradation for the investigated neat PUF and the nanocomposites was also studied. The obtained results revealed the excellent possibility of using the PUF/rGO/ZnO nanocomposite as a superior photocatalyst for the removal of MB dye from wastewater resources. 

## 2. Results and Discussion 

### 2.1. X-ray Diffraction

**Figure 1:**
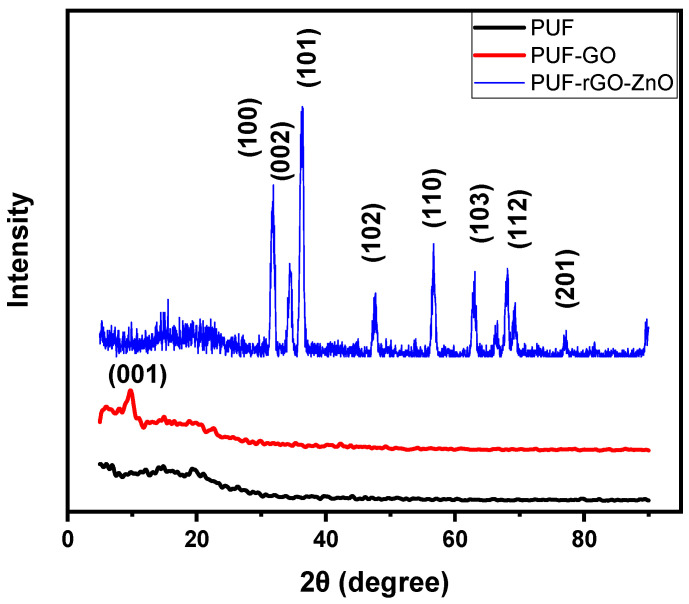
XRD patterns of neat PUF, PUF/GO, and PUF/rGO/ZnO nanocomposites.

XRD was performed to investigate the impact of nanoparticles on the morphological structure of PUF. Figure 1 illustrates the XRD patterns of native PUF and shows the features of the fabricated PUF/rGO, as well as PUF/rGO/ZnO samples with 2θ ranges from 5° to 90°. It was found that a relatively wide diffraction peak appeared at 2θ ~ 20.80°, which was assigned to the amorphous structure of the PUF [35,36,37]. After GO nanosheets were added to the PUF matrix, a relatively sharp diffraction peak appeared at 2θ ~ 9.7^o^, corresponding to the (001) basal plane of GO, when compared to 2θ = 11.40° for native GO nanosheets [38]. Meanwhile, the peak intensities corresponding to PUF were reduced. These results demonstrate that the PUF chains were penetrated between GO nanosheets and expanded the interlayer spacing, leading to the formation of exfoliated or intercalated nanocomposites. For the PUF/rGO/ZnO nanocomposites, new sharp peaks were located at 2θ values 31.8°, 34.4°, 36.3°, 47.5°, 56.6°, 62.9°, 67.9°, and 77°, corresponding to the diffraction planes of (100), (002), (101), (102), (110), (103), (112), and (201), respectively. These sharp peaks are attributed to the hexagonal wurtzite structure of ZnO NPs [39,40,41]. These results agree with those reported elsewhere [42]. The XRD analysis confirms the successful formation of the PUF/rGO/ZnO nanocomposite. 

The diffraction peaks are consistent with card No. 01-078-3322 which has been assigned to ZnO NPs. It was observed that the diffraction peaks of GO nanosheets disappeared, confirming the reduction of GO. Moreover, impurities were not detected in the diffraction patterns, supporting the purity of the synthesized structure. The average crystallite size of ZnO NPs was estimated from the well-known Scherrer equation [35]

(1) $$D\;=\left[\frac{k\lambda }{\beta_{D}\;\cos\;\theta }\right]$$

where *D* is the average crystallite size in nm, *k* is the shape factor, *β_D_* is the full width at half maximum (FWHM), and *θ* is the diffraction angle. The estimated average crystallite size was found to be about 15 nm. 

**Figure 2:**
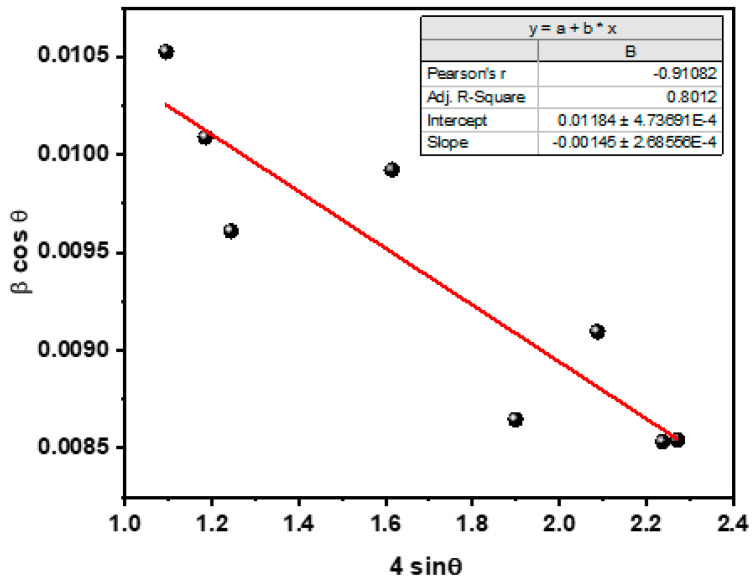
The W-H plot of the PUF/rGO/ZnO nanocomposite.

Figure 2 shows a W-H plot, a mathematical expression for estimating the crystallite size, micro-strain, and dislocation density, using the following equation:

(2) $$\left\{\left(\beta \;\cos\;\theta \right)=\left(4\varepsilon \;\sin\;\theta \right)+\left[\frac{k\lambda }{D}\right]\right\}$$

(3) ε= β cos θ\4

(4) δ = 1D2

The dislocation density was estimated to be 0.004598, while the micro strain was 0.00606. 

### 2.2. FT-IR Analysis 

**Figure 3:**
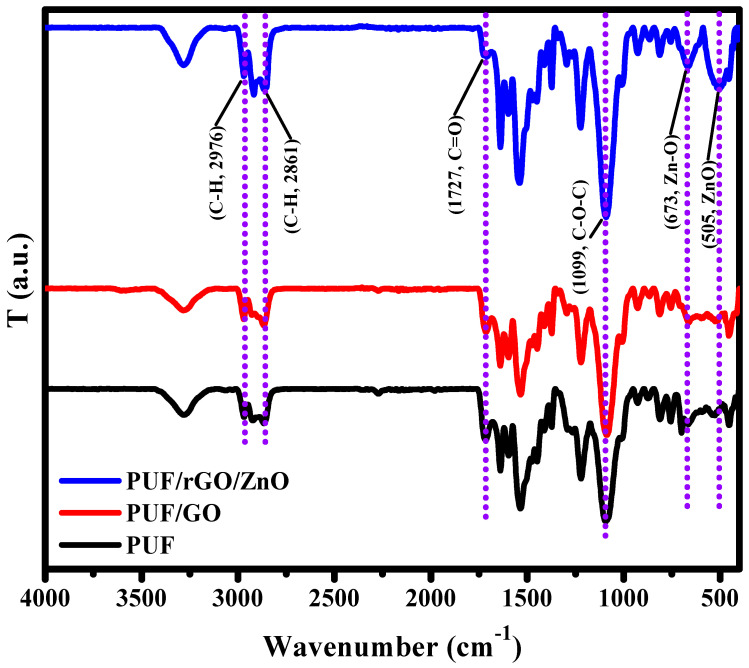
FTIR spectra of neat PUF, PUF/GO, and PUF/rGO/ZnO nanocomposites.

Polyurethane is a type of polymer composed of a repeating urethane bond (-NHCOO-) that can be manufactured through the polymerization of isocyanates and polyols. The FT-IR spectra for the fabricated nanocomposites were recorded with a spectral range from 4000 cm^−1^ up to 450 cm^−1^, as displayed in Figure 3. Both spectra showed absorption bands located at 2976 cm^−1^ and 2861 cm^−1^ (relating to the asymmetric stretching vibrations of C-H in CH_3_, and symmetric vibrations of C-H in CH_2_, respectively) [43] and 1727 cm^−1^ (stretching vibration of C=O). A characteristic band at about 1099 cm^−1^ was assigned to the stretching vibration of C-O-C groups [44]. However, the intensities of the bands at 1727 cm^−1^ and 1099 cm^−1^ decreased when ZnO NPs were added to the PUF/GO matrix, indicating that a chemical interaction of ZnO NPs and the PUF/GO matrix was taken place. Furthermore, two other prominent features of the PUF/rGO/ZnO nanocomposites were observed at 673 cm^−1^ and 505 cm^−1^ and assigned to the ZnO bending vibration modes of ZnO NPs [45]. 

### 2.3. Scanning Electron Microscopy (SEM)

**Figure 4:**
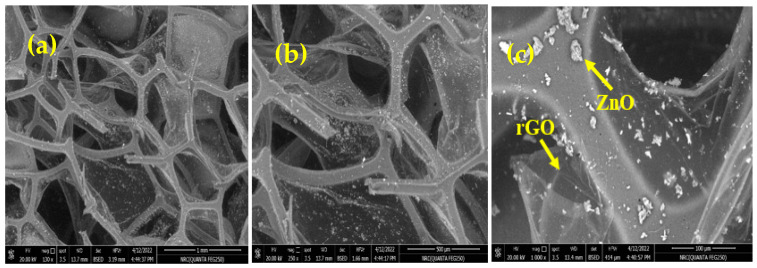
SEM micrographs of PUF/rGO/ZnO nanocomposite (**a**) at low magnification, (**b**) at mid magnification, and (**c**) at high magnification.

**Figure 5:**
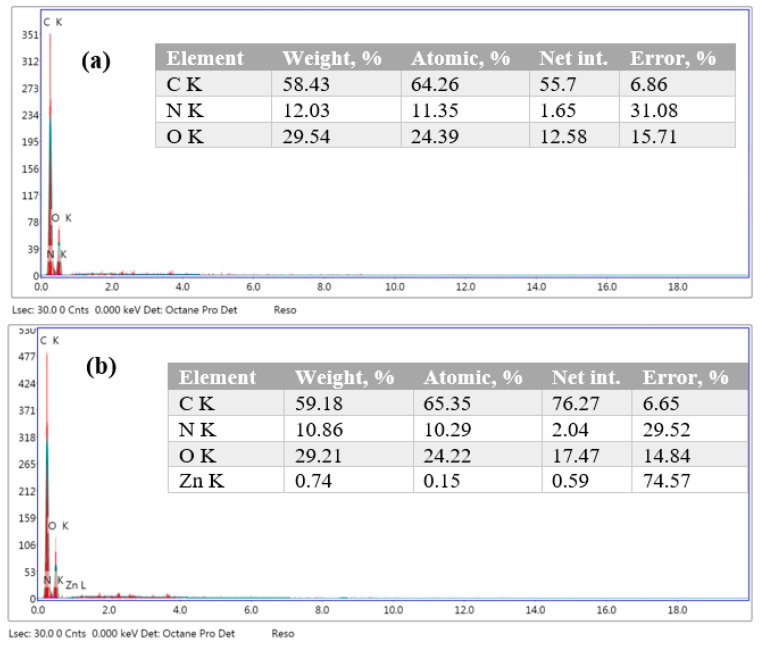
EDX spectra of (**a**) neat PUF and (**b**) PUF/rGO/ZnO nanocomposite.

SEM analysis can reveal valuable information about the embedding of rGO/ZnO NPs and their dispersion quality within the PUF matrix. SEM micrographs representing fractured surfaces for PUF/rGO/ZnO nanocomposites are displayed in Figure 4. For better visualization, three different magnifications are provided. As shown in Figure 5, a fine and more uniform cellular morphology structure was obtained in the nanocomposite matrix. This was due to the embedding and homogeneous dispersion of rGO/ZnO NPs. The higher magnification image shows that the rGO nanosheets appear as wrinkled sheets deposited over the PUF with good adhesion [46]. This leads them to fill the pores of the PUF or trapped between large open pores. On the other hand, the morphology of ZnO NPs appeared as spherical structures, which were well dispersed in the matrix. The EDX measurements were conducted to verify the presence of different elements in the prepared PUF/rGO/ZnO nanocomposite, as depicted in Figure 5. EDX of neat PUF was also taken as a reference. EDX results showed the presence of nitrogen, oxygen, and carbon, which represent the main building block elements of PUF. When zinc oxide and rGO were deposited over a PUF skeleton, an additional peak for Zn was observed in the EDX spectrum, indicating the successful incorporation of zinc oxide in addition to the rGO and PUF. 

### 2.4. The Scanning Transmission Electron Microscope (STEM):

**Figure 6:**
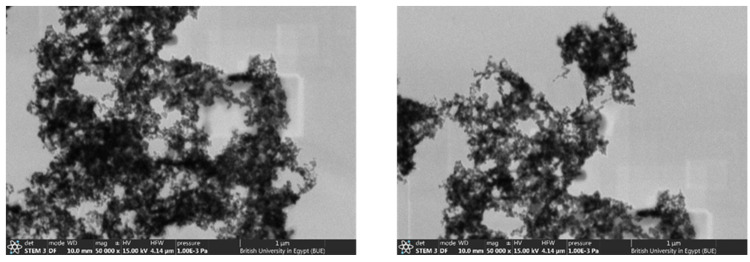
The STEM micrograph of the ZnO/rGO nanocomposite.

STEM is a valuable technique used mainly to investigate fine microstructure materials. In this study, STEM was used to obtain information about the microstructure of the rGO/ZnO nanocomposite. The same experimental procedure was traced as reported above, except for the absence of the PUF. The microstructure of the rGO/ZnO is displayed at different magnifications in Figure 6. The ZnO can be seen to be agglomerated in a dense manner. A more detailed micrograph reveals that the morphology of the ZnO has a semi-regular spherical and rod-shaped structure. 

### 2.5. Surface Area Measurements for Nanoparticles

**Figure 7:**
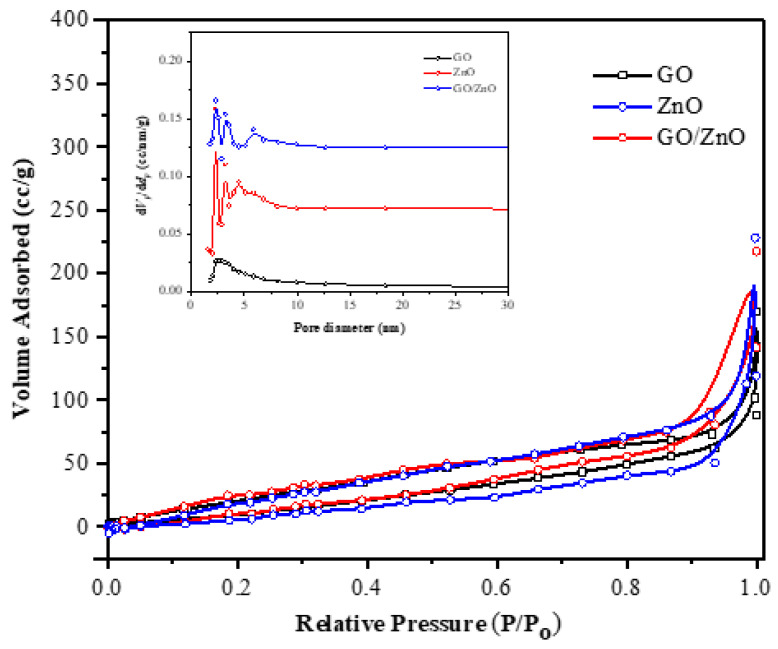
BET-specific surface area for synthesized GO, ZnO, and rGO-ZnO NPs.

The Brunauer–Emmett–Teller (BET) surface area technique was used to determine the surface area of the synthesized NPs. To evaluate the surface area of the fabricated samples, the isotherm results of the ZnO, GO, and GO-ZnO samples are presented in Figure 7, and the data are summarized in Table 1. It was observed that all nanocomposites displayed a type IV isotherm with an H-1 hysteresis loop [47]. Furthermore, the BET-specific surface area for ZnO NPs was low (2 m^2^/g) (Table 1). However, after incorporating the GO, the specific surface area of the ZnO/rGO composite increased to 30.8 m^2^/g. These results may be due to the addition of GO, which has larger pore sizes and surface area. 

### 2.6. UV–Visible Measurements 

**Figure 8:**
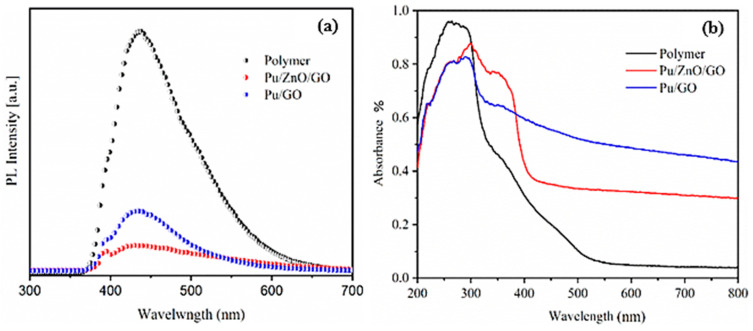
(**a**) UV−vis spectra for neat PUF, PUF/GO, (**b**) PUF/GO/ZnO nanocomposite.

**Figure 9:**
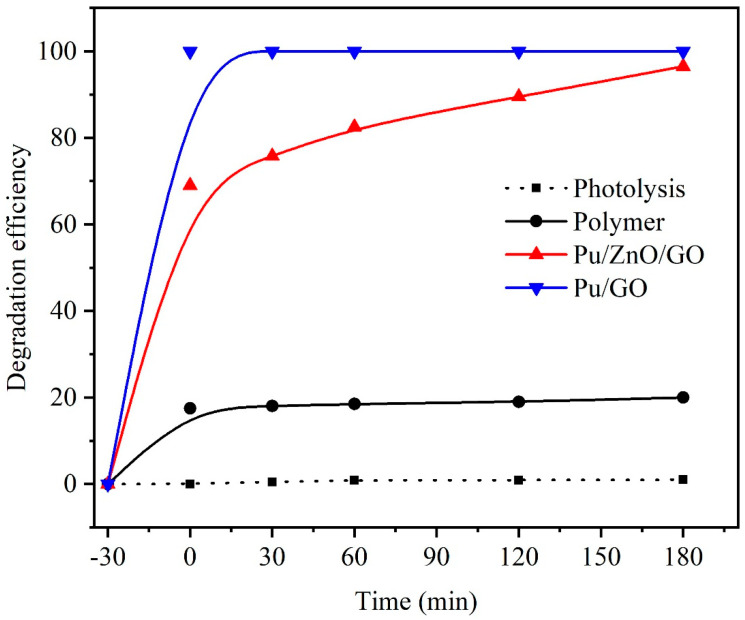
Photocatalytic activity of neat PUF, PUF/GO, and PUF/rGO/ZnO nanocomposites.

UV–visible diffuse reflectance spectrum of the synthesized samples was measured to determine the effect of the presence of ZnO and rGO on the optical properties of PUF, as demonstrated in Figure 8. It was clearly found that the nanocomposite displayed reflectance in the visible regions. The rGO/PUF sample showed a broad absorption in the whole region. The enhanced light harvesting capability of the rGO/PUF sample was attributed to the synergistic effects of the rGO nanosheets and the improved light absorption of rGO sheets [48]. The fluorescent spectra of the PUF, GO/PUF, and PUF/GO/ZnO samples are shown in Figure 9b. As can be seen, the fluorescence spectrum of PUF displayed a broad band emission peak at ∼440 nm. In contrast, the fluorescence for the PUF/GO/ZnO sample was almost significantly quenched. It has been demonstrated that the recombination of photogenerated carriers of ZnO can be effectively suppressed in the presence of carbon materials. The interfacial connections between ZnO and carbon materials reduce the probability of recombination and lead to increased charge carrier separation. In addition, the polymer behaves as a confinement agent.

### 2.7. Photocatalytic Activity

The photocatalytic activity of the synthesized NPs for the degradation of water contaminants under visible light irradiation can be examined using MB dye as a model organic pollutant. Figure 9 demonstrates the photodegradation efficiencies of MB dye in the presence of neat PUF, PUF/GO, and PUF/rGO/ZnO nanocomposites. It is worth mentioning that polyurethane has a high porosity. Thus, adsorption capacity is a critical factor in the efficiency of the photocatalytic process. About 20% of the MB dye was adsorbed on the PUF surface without light. This adsorption capacity was significantly affected by the incorporation of GO into the PUF matrix. As displayed in the figure, the MB dye was completely adsorbed on the PUF/GO sample surface. However, after loading the ZnO NPs in the PUF/rGO/ZnO sample, the adsorption capacity for MB dye initially degraded to approximately 70%. However, 100% degradation was achieved after 3 h. This achievement was probably due to the highly efficient photocatalytic property of rGO/ZnO, therefore increasing the photodegradation efficiency [49]. 

**Figure 10:**
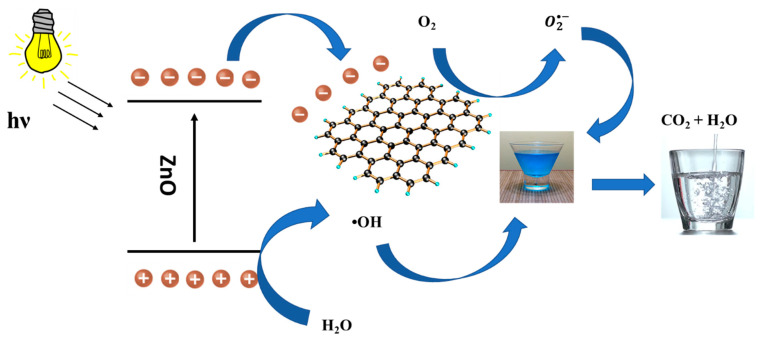
Schematic representation of the photodegradation mechanism.

The proposed structure has many advantages due to the presence of the functional constituents represented in ZnO and rGO, as well as the porous nature of polyurethane foam. It is well known that rGO plays a key role in enhancing photodegradation efficiency. This is attributed to its ability to reduce the recombination of generated electron–hole pairs. Additionally, the presence of defects in the ZnO can also generate extra holes. ZnO is an n-type semiconductor with a band gap of 3.22 eV, and thus visible light cannot excite electrons from the valence band (VB) to the conduction band (CB) [50]. On the contrary, rGO has a small band gap (1.0–1.6 eV), so visible light can excite an electron and cause it to jump from the VB to the CB, leaving a hole behind in the VB. The combination of rGO and ZnO serves as an efficient structure for the degradation of MB [31]. In addition to the presence of the functional ZnO/rGO composite, the 3D polyurethane foam has been selected to act as a scaffold for depositing the composite due to its high absorption capacity and porosity. A photodegradation mechanism can be proposed based on the successful generation and inhibited recombination of electron/hole pairs. When ZnO is subjected to visible light, an electron and a hole are generated in the CB and VB, respectively. The photogenerated electrons can easily migrate from the ZnO CB to rGO. The transferred electrons then react with dissolved O_2_ in water to produce superoxide (O_2_^•−^) radical anions (Equation (7)). These superoxide radicals rapidly react with water to generate OH ions (OH^¯^). Correspondingly, the generated holes in the ZnO VB reacted with (OH^−^) (Equation (8)) to generate hydroxyl (OH^•^) radicals (Equation (9)). The free radicals (O_2_^•−^ and OH^•^) oxidize the MB molecules to form CO_2_, H_2_O, and other byproducts [8,29,31]. The combination between rGO and ZnO produces reactive species responsible for the degradation of MB dye in tandem with the sorption capabilities of PUF. The reaction mechanism can be described in the schematic diagram of Figure 10 and by the following equations:

(5) $$\mathrm{Zn}+hv\to h^{+}\left(\mathrm{CB}\right)+e^{-}\left(\mathrm{VB}\right)$$

(6) $$\mathrm{Zn}\;\left(e^{-}\right)+\;\mathrm{rGO}\;\to \;\mathrm{ZnO}\;+\;\mathrm{rGO}\;\left(e^{-}\right)$$

(7) $$\mathrm{rGO}\;\left(e^{-}\right)\;+\;O_{2}\to \;O_{2}^{•-}+\;\mathrm{rGO}$$

(8) $$O_{2}^{•-}+\;H_{2}O\to \;•\mathrm{OH}\;+\;{\mathrm{OH}}^{-}$$

(9) $$\mathrm{ZnO}\left(h^{+}\right)+\;{\mathrm{OH}}^{-}\to \;\mathrm{ZnO}\;+•\mathrm{OH}\;$$

(10) $$•\mathrm{OH}\;+\;\mathrm{organic}\;\mathrm{pollutant}\;\to \mathrm{degradation}\;\mathrm{products}\;\left(\mathrm{including}\;{\mathrm{CO}}_{2}+\;H_{2}O\right)\;$$

## 3. Materials and Methods

### 3.1. Materials

Polyurethane foam (PUF) with an apparent density of 12–15 kg/m^3^ (97%) was purchased on October 6 from FOAMHOUSE Co., Giza, Egypt. Graphene oxide (GO) was prepared by following the improved Hummer method with slight modifications. The detailed synthesis procedure can be found in previous reports [7,8]. Zinc acetate dihydrate (≥99%) was purchased from Oxford, ammonium hydroxide solution (33%) was purchased from Thermo Fisher Scientific, and granular sodium hydroxide (99%) was purchased from Sigma Aldrich. Triethanolamine (TEA) (98%) was purchased from LOBA Chemie, and commercial grade ethanol (97%) and acetone (90%) were purchased from Al-Nasr Pharmaceutical Co., Cairo, Egypt. Deionized water (DIW) was supplied and used through all experimental work. All commercially obtained chemicals and reagents were used as received; without any further purification, ZnO NPs were prepared using the co-precipitation method. In a typical method, 1 M zinc acetate dihydrate was added to 100 mL DIW and heated to 70 °C, followed by a dropwise addition of 2 M NaOH with stirring for 1 h. Next, the precipitate was collected by centrifugation at 8000 rpm and washed several times with DIW to remove any unreacted starting material. The obtained precipitate was then dried in a laboratory oven overnight at 80 °C, calcined at 550 °C for 2 h, and stored in a desiccator [51,52].

### 3.2. Fabrication of PUF/rGO-ZnO Nanocomposites

**Figure 11:**
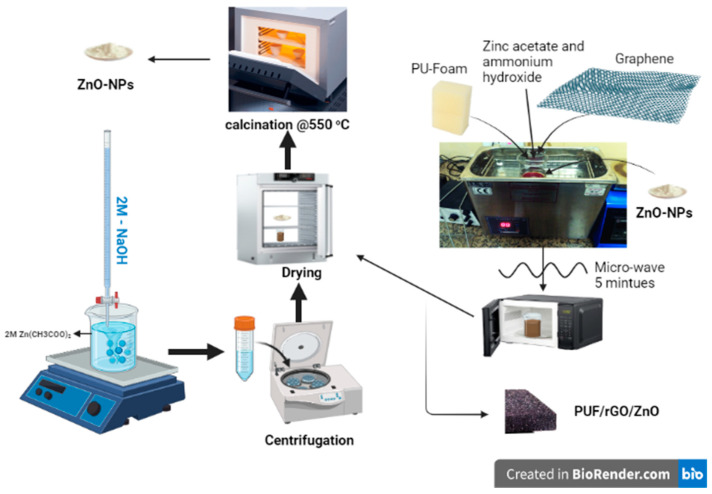
The schematic representation of the synthesis procedure.

The polyurethane foam (PUF) was washed following the standard tabulated procedures. A 20 mm × 20 mm piece was cleaned in acetone in an ultrasonic bath for 10 min before being soaked in DIW for another 10 min. The PUF sample was then dried in a vacuum oven overnight. The cleaned and dried PUF was immersed in a suspension of rGO suspension (3 mg/mL) and squeezed several times. The pressure inside the vessel containing the PUF/rGO suspension was then reduced in order to remove captured air bubbles and force the rGO suspension inside the internal pores of the PUF. This last step was repeated three times to ensure the full coverage of the PUF with GO. To complete this step, the PUF/GO was rinsed in DIW and then dried in a vacuum oven until completely dry. The deposition of ZnO NPs over PUF/GO was achieved by two successive impregnation and hydrothermal processes. The first process consisted of seed layer deposition, while the latter included ZnO growth. The seed layer was deposited by soaking PUF/rGO in a solution of zinc acetate and ammonium hydroxide (pH = 8.5) for 1 h, followed by microwave irradiation for 5 min. The ZnO NPs were grown by hydrothermal methods. The PUF/rGO was soaked for 1 h in a solution of 1.3 mL triethanolamine and 2.1 g zinc acetate before being sealed in a Teflon-lined autoclave and treated in a cycling oven at 100 °C for 4 h. Afterwards, the PUF/rGO/ZnO sponge was washed by DIW for several times and dried in a vacuum oven at 60 °C for 2 h. The schematic diagram of the synthesizing procedure is represented in Figure 11.

### 3.3. Characterization Techniques

The prepared rGO/ZnO@PUF samples were characterized using X-ray diffraction (XRD) using a Malvern Panalytical Empyrean 3 diffractometer to determine the phase composition and crystal structure with step sizes in 2θ = 5. Field-emission scanning electron microscopy (FE-SEM), energy-dispersive X-ray (EDX), and EDX-mapping were used to study the morphology, the elemental composition, and the elemental distribution on the surface of the nanoparticles’ structure using a Quattro S FE-SEM (Thermo Scientific, Waltham, MA, USA) at 20 kV. The Fourier transform infrared (FT-IR) spectra were measured using a Vertex 70 FT-IR spectrometer (Bruker, Germany); the spectra were recorded in a spectral range of 4000–400 cm^−1^ with a spectral resolution of 4 cm^−1^. The bandgap energy (*E*_g_) was calculated using a reflection spectra plot ranging between 200 and 1400 nm, obtained using a JASCO V-770 UV–visible spectrophotometer. A Shimadzu RF-5301PC spectrofluorophotometer with a 150 W xenon lamp as an excitation source was used to measure the PL spectra of the samples and solutions at room temperature. The mean values of surface area and pore size distribution were calculated from the adsorption branch based on the BJH model using the BELSORP MAX II program.

## 4. Conclusions

In the present research, a 3D polyurethane foam loaded with rGO and ZnO nano- particles has been synthesized using a hydrothermal route followed by microwave irradiation. XRD measurements demonstrate the successful loading of rGO/ZnO over the PUF scaffold. The average crystallite size was estimated from XRD to be 15 nm. The elemental analysis using EDAX confirmed the presence of the constituent elements Zn, O, and C. The UV–visible measurements demonstrate that the PUF/rGO/ZnO nanocomposite displays reflectance in the visible regions. The photodegradation measurements demonstrate that about 20% of the MB dye was adsorbed on the PUF surface without light. This adsorption capacity was significantly affected by the incorporation of GO into the PUF matrix. As displayed in the figure, the MB dye was completely adsorbed on the PUF/GO sample surface. Interestingly, after incorporating the ZnO NPs into the PUF/rGO/ZnO sample, the adsorption capacitance of the MB dye has shown approximately 70% of initial degradation. However, this degradation increased over time to reach 100% degradation after 3 h.

## Figures and Tables

**Table 1:** Surface area parameters obtained from BET surface area analyzer.

NPs Name	Surface Area m^2^/g	Average Pore Diameter nm	Total Pore Volume cm^3^ g^−1^
ZnO	2.2	4.3	0.13
GO	71.5	13.2	0.20
GO/ZnO	30.8	56.2	0.169

## Data Availability

All the generated data are enclosed within the manuscript.
